# Insights on the evolution of trehalose biosynthesis

**DOI:** 10.1186/1471-2148-6-109

**Published:** 2006-12-19

**Authors:** Nelson Avonce, Alfredo Mendoza-Vargas, Enrique Morett, Gabriel Iturriaga

**Affiliations:** 1 Centro de Investigación en Biotecnología-UAEM, Av. Universidad 1001, Col. Chamilpa, Cuernavaca 62210, Mexico; 2 Instituto de Biotecnología-UNAM, Av. Universidad 2001, Col. Chamilpa, Cuernavaca 62210, Mexico

## Abstract

**Background:**

The compatible solute trehalose is a non-reducing disaccharide, which accumulates upon heat, cold or osmotic stress. It was commonly accepted that trehalose is only present in extremophiles or cryptobiotic organisms. However, in recent years it has been shown that although higher plants do not accumulate trehalose at significant levels they have actively transcribed genes encoding the corresponding biosynthetic enzymes.

**Results:**

In this study we show that trehalose biosynthesis ability is present in eubacteria, archaea, plants, fungi and animals. In bacteria there are five different biosynthetic routes, whereas in fungi, plants and animals there is only one. We present phylogenetic analyses of the trehalose-6-phosphate synthase (TPS) and trehalose-phosphatase (TPP) domains and show that there is a close evolutionary relationship between these domains in proteins from diverse organisms. In bacteria TPS and TPP genes are clustered, whereas in eukaryotes these domains are fused in a single protein.

**Conclusion:**

We have demonstrated that trehalose biosynthesis pathways are widely distributed in nature. Interestingly, several eubacterial species have multiple pathways, while eukaryotes have only the TPS/TPP pathway. Vertebrates lack trehalose biosynthetic capacity but can catabolise it. TPS and TPP domains have evolved mainly in parallel and it is likely that they have experienced several instances of gene duplication and lateral gene transfer.

## Background

One of the fundamental challenges for an organism is to survive changes in the physical environment-mainly extreme temperatures, salinity, or dehydration. This problem was to be solved very early in evolution since the first cells inhabited the primitive seas [1,2]. Organisms evolved two different strategies to contend with abiotic stress. In certain species that live in extreme environments, for instance strict thermophiles and halophiles, the metabolic capabilities were modified, such that the optimal enzymatic activity or membrane stability are at high temperature or salinity, respectively [3]. Other organisms when exposed to extreme conditions have a drastically different adaptation to contend with stress. They evolved biosynthetic pathways for osmotically active compounds, cryoprotectants or thermoprotectants, thus enabling survival until conditions are favourable again. Among these compounds are polyols such as mannitol, sorbitol, some amino acids (proline and glutamic acid); quaternary ammonium salts, for instance glycine betaine; and disaccharides, for example sucrose and trehalose [4]. This latter compound is a non-reducing disaccharide formed by two glucose molecules linked by a 1α-1α bond which is present in several organisms and common foodstuffs such as bread, wine, beer, vinegar, and honey [5].

Many functions have been described for trehalose-for instance in prokaryotes trehalose is frequently used as a compatible solute to contend with osmotic stress and can be used as an external carbon source [6-8]. In bacteria of the genera *Mycobacteria*, *Nocardia*, *Rhodococcus *and *Corynebacterium*, trehalose is present in the cell wall glycolipids [8-10]. In yeast, trehalose can be used as a reserve compound [11,12] and for the adaptive response to different types of abiotic stress [8,13-16]. Also, it has been shown that in yeast trehalose 6-phosphate, the trehalose biosynthesis intermediate is a regulator of the glucose metabolic flux during glycolysis [17-20].

In several organisms trehalose is capable of stabilising and protect membranes and proteins, allowing anhydrobiotic organisms to survive cycles of dehydration-rehydration [21,22]. In insects trehalose is the most abundant sugar in the haemolymph (80–90%) and in thorax muscles, were it is consumed during flight [10,23]. Until recently, it was thought that in plants trehalose was only synthesised in the so-called "resurrection" plants such as *Selaginella lepidophylla *and *Myrothammus flabellifolius*, where it is the key molecule to protect against stress, especially drought. However, a large number of studies with transgenic plants, along with the sequencing information available for many plant genomes, suggest that trehalose can be synthesised in several other plants. Furthermore, in *Arabidopsis thaliana *it has been demonstrated that trehalose has a fundamental role in embryo development [24], and in abscisic acid and sugar signalling [25].

**Figure 1:**
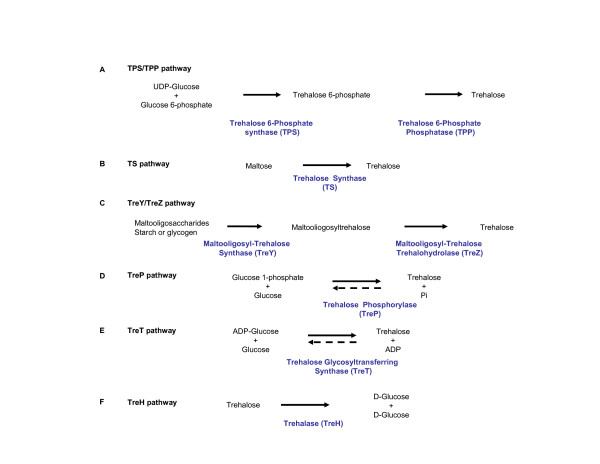
**The trehalose biosynthetic pathways**. The enzymes are indicated in blue.

There are at least five biosynthetic pathways known for trehalose (Figure 1). The first pathway was discovered about 50 years ago [26], is the most widely distributed, and it has been reported in eubacteria, archaea, fungi, insects, and plants. It involves two enzymatic steps catalyzed by trehalose-6-phosphate synthase (TPS) and trehalose-phosphatase (TPP). TPS catalyzes the transfer of glucose from UDP-glucose to glucose 6-phosphate forming trehalose 6-phosphate (T6P) and UDP, while TPP dephosphorylates T6P to trehalose and inorganic phosphate [5,9] (Figure 1A). In the second biosynthetic pathway, the enzyme trehalose synthase (TS) isomerises the α1-α4 bond of maltose to a α1-α1 bond, forming trehalose [5,27]. This enzyme was first reported in *Pimelobacter sp*. and orthologs of this protein have been found in other eubacteria (Figure 1B). The third pathway involves the conversion of maltodextrines (maltooligosaccharides, glycogen and starch) to trehalose. This pathway was reported in thermophilic archaea of the genus *Sulfolobus*. These organisms synthesize trehalose in two enzymatic steps catalyzed by maltooligosyl trehalose synthase (TreY), coded by the *treY *gene, which promotes the transglycosylation of the last glucose moiety at the reduced end of maltodextrins from a α1-α4 to a α1-α1 bond leading to maltooligosyltrehalose, which contains a trehalose moiety at the end of the polymer. Next, maltooligosyl trehalose trehalohydrolase (TreZ), coded by the *treZ *gene, catalyses the hydrolytic release of trehalose (Figure 1C) [5,28]. In the fourth pathway, trehalose phosphorylase (TreP), present in some fungi, catalyses the reversible hydrolysis of trehalose in the presence of inorganic phosphate. The transfer of a glucose molecule to a phosphate generates glucose 1-phosphate and releases the other glucose residue. There is uncertainty about the participation of the TreP enzyme in the synthesis or degradation of trehalose, since the biosynthetic reaction has only been shown *in vitro *[29,30] (Figure 1D). A new biosynthetic pathway for trehalose was found in the hyperthermophilic archaeon *Thermococcus litoralis*, and involves the trehalose glycosyltransferring synthase (TreT), which catalyses the reversible formation of trehalose from ADP-glucose and glucose [31,32]. It can also use UDP-glucose and GDP-glucose, although it is less efficient with these substrates. The TreT enzyme transfers the glucose moiety from ADP-glucose, and joins it at position 1 of another glucose molecule to form trehalose (Figure 1E). Trehalose is degraded by trehalase (TreH) into two glucose molecules (Fig. 1F).

In this work, the presence in the completely sequenced genomes of genes coding for the trehalose biosynthetic enzymes was analysed. We found that these enzymes are widely distributed in the three domains of life and moreover, many organisms have more than one, and sometimes several pathways for trehalose synthesis. Also, we inferred the phylogenetic relationships of TPS and TPP enzymes. Interestingly, we found that they have significantly coevolved. This is supported by the fact that in prokaryotes the genes coding for these enzymes are generally organised as a single operon, while in eukaryotes there are multigene families, generally each member coding for a fused polypeptide with TPS and TPP domains. The functionality of many of these genes is supported by the observation that the catalytically relevant residues for both TPS and TPP are highly conserved.

## Results and discussion

### Distribution of trehalose pathways

**Figure 2:**
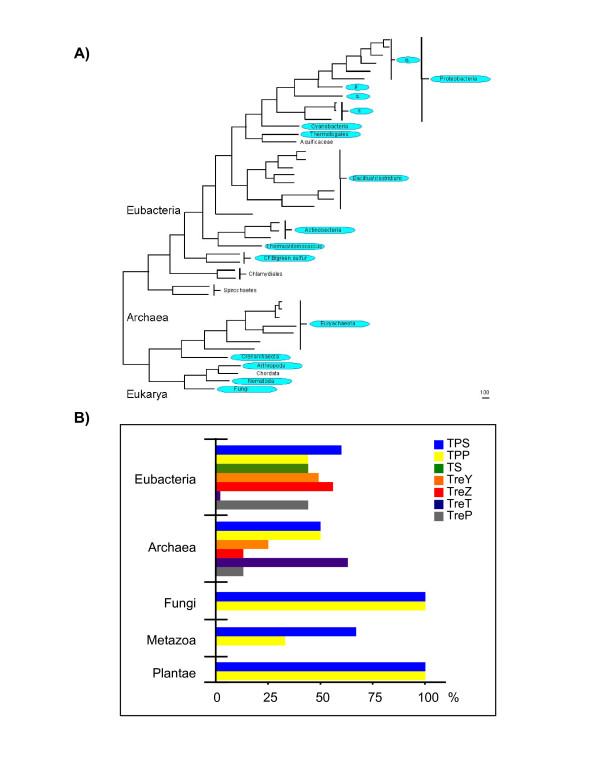
**The distribution of trehalose biosynthetic proteins in nature**. (A) The phyla with al least one synthesis pathway. (B) Comparative analysis between the percentage of completely sequenced genomes with at least one biosynthetic pathway.

To identify genes involved in the five known pathways for trehalose biosynthesis, we carried out BLAST searches [33] using the amino acid sequences from enzymes of several organisms with the NCBI non-redundant databases of proteins. Homologues with significant scores (expect value >E^-9^, see Methods) were identified in a wide range of organisms including archaea, eubacteria, plants, fungi, and animals (Figure 2A).

Information from the completely sequenced genomes allowed us to identify the presence of each of the five different trehalose biosynthesis pathways in individual organisms. For this study 205 genomes were analysed, 171 from eubacteria, 18 from archaea, 8 from fungi, 3 from mammals, 2 from plants, 2 from insects and one from a nematode. Selected sequences for further analysis are shown in Additional file 1. Since the number of sequenced genomes is not uniform for each taxonomic group, the presence of trehalose biosynthetic protein domains is shown as the percentage value related to the total genomes with at least one pathway (Figure 2B).

Prokaryotes showed the greatest distribution of trehalose pathways (see Additional file 1). Eubacteria was the only group where the five pathways were found although not all together in a single species, most of them having from two to four pathways. The presence of several biosynthetic pathways in the same organism may be due to the strict requirement to accumulate trehalose under changeable environmental conditions, which could limit substrate availability for each pathway. A number of eubacterial species have multiple copies of some of these pathways. For example, *Mesorhizobium loti*, *Mycobacterium tuberculosis*, *Ralstonia solanacearum *and *Xanthomonas campestris *have two *TPS *genes, while *Thermoanaerobacter tengcongensis *has two genes for trehalose phosphorylase (*treP*). *M. loti *has a truncated copy of TPS, which we excluded from analyses. Phylogenetic analyses suggest that paralogous TPS genes are the product of lateral gene transfer events instead of recent gene duplications (see below). Interestingly, the TS pathway might be exclusive to eubacteria, while TreT was only observed in Archaea and *Thermotoga maritima*, a thermophilic eubacteria which inherited a substantial part of its genome from Archaea by lateral gene transfer [34]. In fungi, plants and invertebrates only the TPS/TPP pathway is present; no homologues to other trehalose biosynthetic genes were found. Interestingly, none of the five described trehalose biosynthesis pathways was found in vertebrates, although the presence of TreH, the trehalose catabolic enzyme, has been reported in this group of organisms [5].

The above analyses show that trehalose biosynthesis pathways have a patchy distribution across biological groups, ranging from the coexistence of several pathways in eubacterial species, to a single pathway in eukaryotes. Plants contain multiple TPS/TPP sequences. It would be interesting to explore the functionality of these reiterated gene copies.

### Functional adaptation of TPS/TPP enzymes

In order to address the functionality of the multiple copies of eukaryotic TPS/TPP genes, we analysed whether the relevant amino acid positions are conserved in these proteins (see Additional file 2). The reported 3D structure of the *E. coli *TPS enzyme allowed the identification of the amino acid residues involved in the binding of substrates and in catalysis [35]. The residues involved in the binding of glucose 6-phosphate are Arg9, Trp40, Tyr76, Trp85 and Arg300, while Gly22, Asp130, His154, Arg262, Asp361 and Glu369 are involved in the binding of UDP-glucose [35]. Interestingly, several proteins of Arabidopsis and yeast that do not complement a *ΔTPS1 *mutant of *S. cerevisiae *[36,37] lack several of these residues. In contrast, all these residues are conserved in proteins displaying TPS activity so far reported, or from organisms that are known to produce trehalose (Additional file 3). There are eight *TPS *genes in Arabidopsis but only AtTPS1 and AtTPS2 proteins have these residues, and the enzymatic activity has only been demonstrated for AtTPS1 [38]. None of the TPS sequences of *Oryza sativa *have these residues conserved and we did not find any other gene of an alternative trehalose biosynthetic route in this organism (see additional file 2). However, trehalose is accumulated in rice upon salt stress [39].

The sequence analysis of plant and fungal TPS proteins, which do not have the conserved positions, showed that they have been selected only with very limited substitutions. One of the most prominent changes is R262D in *A. thaliana *and *O. sativa *versus R262Q/K in *S. cerevisiae *and *S. pombe*; W40Y/F was only found in plants.

TPP belong to the HAD (L-2-haloacid dehalogenase) superfamily of magnesium-dependent phosphatases/phosphotransferases, which is distributed in both prokaryotes and eukaryotes [40,42]. This superfamily uses a common catalytic reaction mechanism characterised by having three highly conserved motifs. Region 1 (motif 1) near to the N-terminal end, contains the DXDX(T/V) sequence, where the first aspartic acid residue forms a phosphorylated intermediate with the substrate, while the second residue plays an important role in catalysis. Region 2 (motif 2) has a conserved serine or threonine (S/T)(GX), which serves to form a hydrogen bond with a phosphate group of the substrate. Region 3 (motif 3) contains the K(X)16–30(G/S)(D/S)XXX(D/N) sequence being part of the active site and coordinates the magnesium ion required for catalysis [40,41]. Recently, the crystalographic structure of TPP from *Thermoplasma acidophilum *was determined [42].

In the present work we asked the question whether the mentioned motifs are conserved within the sequences of the TPP domains. Most TPPs such as OtsB, ScTPS2, AtTPPA-B, and AtTPS5-11 proteins contain the highly conserved residues of HAD superfamily active site in the three conserved regions and also displayed a high degree of similarity in the rest of the protein (see Additional file 4). The data showed significant similarity among archaea, eubacterial, fungi and plant TPP. However AtTPS1 to 4 and 8, *S. cerevisiae*_3 and 4 (ScTPS3 y ScTS11), *S. pombe*_4 and _5, and *E. gossypi*_3 proteins lack these conserved positions (see Additional file 5). Therefore it is unlikely that these proteins could have the phosphatase activity. Similarly, in OsTPS_2 the conserved arginine of motif 2 has been replaced by phenylalanine. The absence of residues directly implicated in catalysis as evidenced by 3D structure imply that these proteins are not phosphatases although the remaining domain is conserved.

### Search of functional TPS isoforms

Since the majority of TPS/TPP fusion proteins lack the ability to complement TPS or TPP deficient yeast strains, it is possible that they are non-functional enzymes. Alternatively, they could have acquired new functions. In order to predict possible functionality of these proteins we determined the selective pressure at the amino acid level for each of the TPS and TPP domains of eukaryotic proteins (Additional file 6). The rate of mutagenesis substitutions, *ω*, is calculated by comparing the ratio of non-synonymous (*dN*) to synonymous (*dS*) substitutions, *ω *= *dN/dS*. If *ω *is negative, it means that the majority of accumulated substitutions at each codon are synonymous, indicating that natural selection is acting upon these positions to preserve functionality (purifying selection). On the contrary, when *ω *value is positive, non-synonymous substitutions have been accumulated at a greater extent, indicating that positive or adaptive selection has taken place [43]. In practice it has been difficult to observe clear cases of positive selection since the early methods averaged *ω *over the entire sequence and in all sequences analyzed. There are refined methods to allow site and lineage specific analysis of codon substitutions [44]. The data obtained with SLAC [45] and PAML [44,46] programs show that practically all codon substitutions found in the DNA sequences encoding both TPS and TPP domains were synonymous at the protein level, *i.e. *the *ω *value was very small for both domains. Only five codons from TPP domain were detected with a positive *ω*, whereas all TPS residues showed a negative value (see Additional file 6). These data indicate that both proteins are under strong purifying selection and, therefore, they must maintain some function, perhaps related to their original enzymatic activity, and that they are not on the process of becoming pseudogenes or are under strong adaptive selection.

### Physical organisation of the TPS/TPP domains

The TPS protein is formed by a single phosphatase domain in prokaryotes while in almost all eukaryotes and in *P. aerophylum *the TPS proteins are fused to the TPP domain. This organization suggests that all the eukaryotic TPS and TPP fused proteins descend from a common ancestor (see Additional file 7). Thus, it is likely that those proteins that do not present this gene fusion, such as *E. gossypii*_1, *S. cerevisiae*_1, *S. pombe*_1 and *E. cuniculi *proteins, have lost its TPP domain. *C. elegans*_1 and *C. elegans*_2 proteins are not fused to TPP and are clustered with Streptomyces apart from the rest of the eukaryotic proteins (see below).

Therefore, we decided to explore if there is a phylogenetic relationship between the TPS and TPP domains, at both the protein structural organization (fusion domains) in eukaryotes, and by the genomic context (gene neighbourhood) in prokaryotes. To analyze the genomic context we used the GeCont server [47], which displays the neighbouring genes of any gene in the fully sequenced genomes [48]. The results found in this study showed a high conservation of the genomic linkage of the TPS and TPP proteins in eubacteria and in archaea, very likely forming part of a single operon. The only exceptions are bacteria of the genus *Mycobacterium*, cyanobacteria and *S. meliloti *in which the TPS and TPP coding genes are not clustered in the genome.

What is the evolutionary significance of TPS and TPP fusion in eukaryotic proteins? Interestingly, although there are eukaryotic proteins comprised by the two domains [49], so far an enzyme with both TPS and TPP activities has not been found. On the other hand, some putative TPS/TPP fusion proteins lack both enzymatic activities and are only regulatory subunits, such as ScTPS3 and ScTSL1 [37]. According to the analysis of TPS and TPP consensus regions, we identified three proteins in *P. aerophilum, D. melanogaster *and *A. gambiae *species, conserving the residues and active sites of both TPS and TPP domains. These are the first putative TPS/TPP fusion proteins with the two domains found so far. An active bifunctional enzyme has been obtained artificially by fusing the *E. coli *OtsA and OtsB domains, to yield a chimerical enzyme with both TPS and TPP activities and trehalose biosynthesis capacity in *E. coli *and *O. sativa *[50,51]. These rice transgenic plants were stress tolerant. Similarly, we constructed a bifunctional TPS/TPP fusion protein using the *S. cerevisiae *domains and it is active both in yeast and *A. thaliana *[unpublished results].

The genomic linkage and fusion in a single polypeptide of TPS and TPP strongly suggest that they have evolved in parallel. To test this hypothesis we performed a phylogenetic analysis of TPS and TPP enzymes.

### Phylogenetic analysis of the TPS proteins

**Figure 3:**
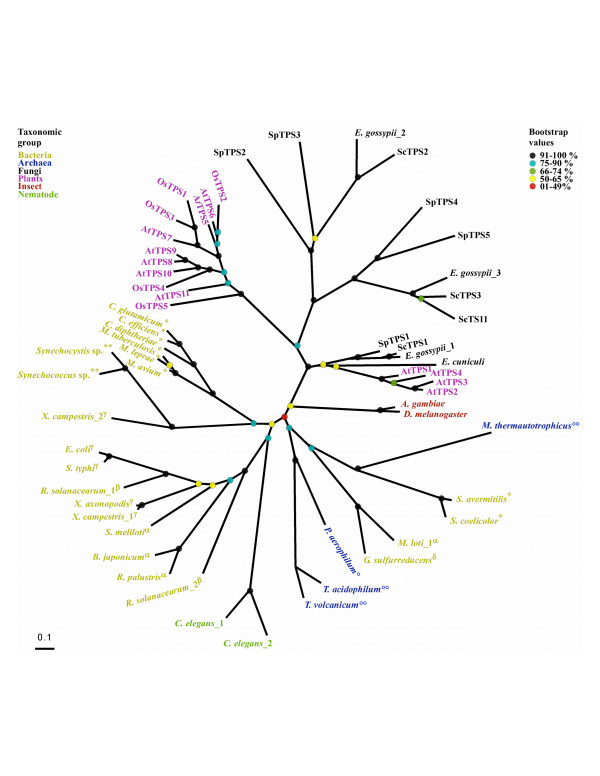
**The phylogeny of the TPS domain**. The tree was generated with the PHYML maximum likelihood software [69]. The proteins are shown in colours according to their taxonomic group. The subdivisions of bacteria are: *Actinobacteria, **Cyanobacteria, ^α ^alpha-proteobacteria, ^β ^beta-proteobacteria, ^γ ^gamma-proteobacteria, ^δ ^delta-proteobacteria. The archaeal groups are: °Crenarchaeota, °°Euryarchaeota. The Bootstrap values (1000×) are shown in percentage.

In order to explore the evolutionary patterns of the TPS and TPP proteins, we performed a comprehensive phylogenetic analysis. Amino acid sequences of TPS coding genes were aligned using CLUSTAL_X [52]. The alignment was edited using the program Seaview [53] and it is shown in the Additional file 2. Phylogenetic reconstructions were carried out using distance based, parsimony and maximum likelihood methods using the amino acid sequences. Tree topologies were fairly consistent with the three methods. The best maximum likelihood tree is shown in Figure 3 with the bootstrapping values for each branch. The TPS sequences were clearly grouped in two major branches: in the first branch are the plant and fungi proteins. This branch is supported by a very high bootstrapping value. The second group comprises the eubacterial and archaeal proteins; the nematode sequences were found within this cluster, suggesting a possible lateral gene transfer event. Insect TPS are in between the two groups. The majority of the clades are supported by high bootstrapping values.

The fungi and plant sequences are clearly arranged into three subgroups: In the first group *Arabidopsis *AtTPS1 to 4 proteins are clustered with *Saccharomyces cerevisiae *(*S. cerevisiae*_1)*, Eremothecium gossypii *(*E. gossypii*_1)*, Schizosaccharomyces pombe *(*S. pombe*_1) and *Eremothecium. cuniculi *(*E. cuniculi*). The second group comprises the rest of the *A. thaliana *proteins and the five *O. sativa *TPS proteins. The third group has the *S. pombe *TPS 2 and 3 (SpTPS2 and 3) proteins and the ScTPS2 and *E. gossypii*_2 proteins. The last group has the rest of the *S. cerevisiae *and *E. gossypii*_3 TPS proteins. This grouping clearly indicates that the ancestor of the plant and fungi had at least two copies of the TPS coding genes, and that there were several episodes of gene duplication within each species, leading to gene copies with high degree of sequence similarity. From the TPS proteins for which functional biochemical evidence exists, only those in the early branching have TPS activity (AtTPS1, ScTPS1, SpTPS1).

Within the prokaryotic group there is a clear branch comprising all the proteobacterial species [alphaproteobacteria (*B. japonicum*, *Rhodopseudomonas palustris*, *Sinorhizobium meliloti*), betaproteobacteria (*R. solanacearum*), gammaproteobacteria (*Escherichia coli*, *Salmonella typhi*, *X. campestris *and *Xanthomonas axonopodis*)]. The actinobacteria (Mycobacterium and Corynebacterium) and members of Cyanobacteria (*Synechocystis sp. and Synechococcus sp*.) phylum are located in a different branch. The TPS protein tree topology is not consistent with standard organismal phylogeny. These inconsistencies arise only for genes displaying multiple paralogous copies in a single organism, suggesting either lateral gene transfer or differential loss of paralogs. For example *X. campestris *has two TPS proteins, *X. caspestris*_1, which is grouped with the rest of the gammaproteobacteria, while the *X. caspestris*_2 is closely related to Cyanobacteria. *M. loti *has also two sequences and it seems that the *M. loti*_1 protein was also recruited by lateral gene transfer, probably after losing almost half of the native gene which codes for *M. loti*_2. The *S. coelicolor *and *Streptomyces avermitilis *TPS were also found apart from the rest of the actinobacteria were they belong. The archaeal TPS are clustered except for *M. thermautotrophicus*, which appears to be more related to actinobacteria of the genus Streptomyces.

### Phylogenetic analysis of the TPP proteins

**Figure 4:**
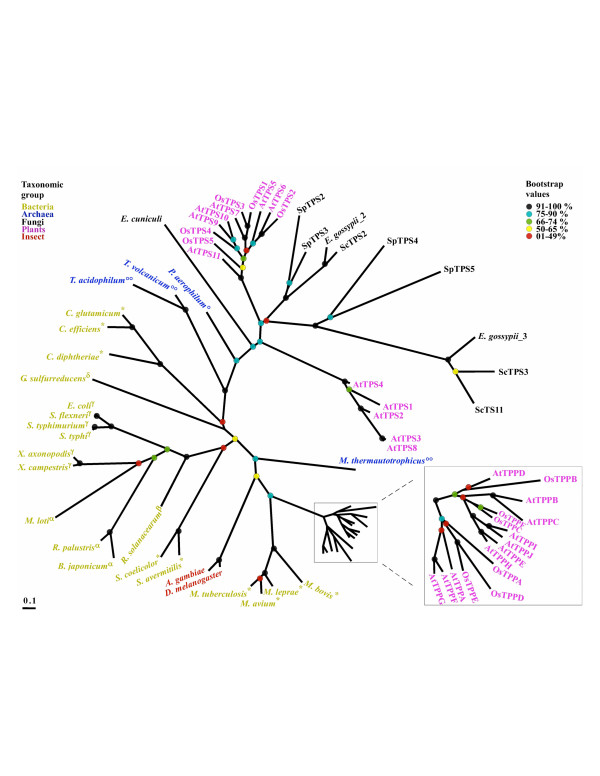
**The phylogeny of the TPP domain**. The tree was generated with the PHYML maximum likelihood software [69]. The proteins and taxonomic subdivisions are shown as in Fig. 3. The Bootstrap values (1000×) are shown in percentage.

From the completely sequenced genomes we obtained 68 sequences with significant sequence similarity to TPP. The sequences of the conserved regions corresponding to the TPP domain were aligned (see Additional file 4), manually edited, and subjected to phylogenetic analyses as described above for TPS. Trees generated by Neighbour Joining, Parsimony and Maximum Likelihood were very similar and Figure 4 shows the best Maximum Likelihood tree. The bootstrapping values of this tree were not as high as those obtained for TPS, nevertheless the fact that the three different phylogenetic reconstruction methods produced similar tree topologies (data not shown) strongly support the general topology.

The phylogenetic tree clearly displays subgroups between the previously described TPP classes in *A. thaliana*: Class I (AtTPS_1–4), Class II (AtTPS_5–11) and Class III (AtTPPA-J) [49,54]. The tree also shows that in *O. sativa *there are only sequences highly related Class II and III. Class I and II display a fused TPS domain and seem be monophyletic with the fungal TPP. As expected, the branching pattern of both TPS and TPP proteins in this group of fused polypeptides is highly similar, except for AtTPS8 that clusters within Class I, perhaps by a gene conversion event with AtTPS3 since these TPP domains are almost identical. Class III comprises small proteins with the TPP domain only. This class of proteins probably was recruited in plants after the divergence from fungi since they are not present in the latter organisms as single domain proteins. It is interesting that class III proteins are closely related to *Mycobacterium *and it is tempting to speculate that they were recruited from bacteria by an ancestor of contemporary plants. Interestingly, from the three TPP classes, only enzymatic activity has been demonstrated for members of this latter Class (III). For instance, AtTPPA and AtTPPB complemented a *S. cerevisiae tps2 *mutant (which lacks TPP) [55]. However, it cannot be discarded that some Class I or Class II TPP proteins could have phosphatase activity as well.

The fungal TPP sequences cluster together except *E. cuniculi*, and apparently they are closer to plant class I and II than to bacterial sequences. Archaeal sequences do not appear as monophyletic. In contrast to bacterial TPS domains, no TPP domain was found duplicated in any single bacterial species.

The TPS and TPP trees displayed in Figures 3 and 4 show similar overall topologies, confirming that there is a high evolutionary correlation between TPP and TPS domains, which was suggested by the fusion of both domains in most of eukaryotic proteins and their clustering in the prokaryotic genomes (see Additional file 8).

### Roles of TPS/TPP homologues in eukaryotes

Why do eukaryotic organisms have several copies of TPS genes? The synthesis and metabolism of trehalose has been studied in many different organisms. In *S. cerevisiae *there are four proteins with TPS and TPP domains, forming a holoenzyme complex where the ScTPS1 and ScTPS2 subunits have a catalytic function while ScTPS3 and ScTSL1 have a regulatory role [37]. In *A. thaliana *the function of the 10 AtTPS1*-*homologues is unknown [49]. The finding in the present work that all *TPS *plant genes (class I, II and III) are under selection pressure suggests that all of them have a particular function, which could probably be related to other processes not necessarily related to osmoprotection. For instance, some studies concerning *AtTPS7 *and *AtTPS8 *genes from Arabidopsis showed that they are unable to complement the *tps1Δ *or *tps2Δ *mutant from *S. cerevisiae *[36] and their products did not have TPS activity. In contrast, *AtTPS1 *and *SlTPS1 *were able to restore growth and trehalose synthesis in these yeast mutants [56,57]. Also, in *A. thaliana *transcription of *TPS *genes is differentially regulated by glucose: *AtTPS1 *[25,58]*AtTPS8*, *AtTPS9 *and *AtTPS10 *are repressed, whereas the expression of *AtTPS5 *increases significantly and the rest of the gene-family members remain without change [59]. It is noteworthy the differential expression of *TPS *genes in response to glucose, suggesting an important role for these genes in the regulation of carbon and sugar metabolism [60]. Overexpression or mutant analyses of the *TPS *genes will uncover their function. In the case of *AtTPS1*, the knockout mutant displays an embryo lethal phenotype, suggesting the role of this gene in plant development [24]. In addition, the overexpression of *AtTPS1 *shed light on its role as a regulator of glucose, abscisic acid, and stress signalling [25].

### Expression pattern of TPS genes in plants

**Figure 5:**
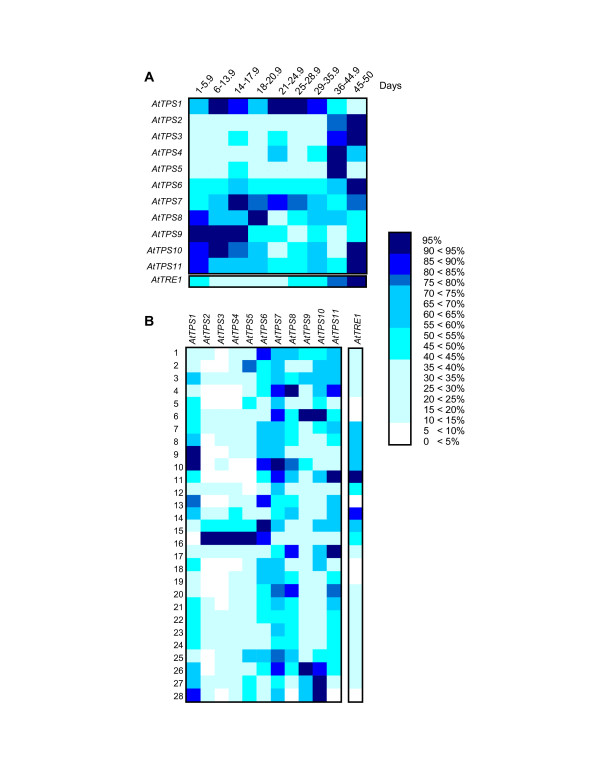
**Expression levels of Arabidopsis *TPS *multigene family**. The figure shows the transcripts level detected in microarrays experiments. (A) Detection along the Arabidopsis life cycle. (B) Organ-specific expression [1 Callus; 2. Cell suspension; 3. Seedlings; 4. Cotyledons; 5. Hypocotyl; 6. Radicle; 7. Inflorescence; 8. Flower; 9. Carpel; 10. Petal; 11. Sepal; 12. Stamen; 13. Pedicel; 14. Silique; 15. Seed; 16. Embryo; 17. Stem; 18. Node; 19. Shoot apex; 20. Cauline leaf; 21. Rosette; 22. Juvenile leaf; 23. Adult leaf, 24. Petiole; 25. Senescent leaf; 26. Roots; 27. Lateral leaf; 28. Elongation zone]. The intensity of the blue colour indicates the level of expression as percentage for each gene, as displayed in the Genevestigator server [61].

To gain further understanding of the *TPS *gene family, the expression pattern of *TPS *genes and trehalase (*AtTRE1*) from *A. thaliana *were analyzed during development and in a tissue-specific manner using the Genevestigator server [61]. Also, the Meta-Analyzer program was used which is designed to analyse the expression of a multi-gene family under different conditions and in different tissues and growth stages. In Figure 5A the expression levels of *AtTPS1 *to *AtTPS11 *and trehalase genes is shown, during the different plant developmental stages. The data showed that all *TPS *genes are expressed in the plant in a particular tissue and specific stage. The *AtTPS1, AtTPS6, AtTPS7 *and *AtTPS11 *genes displayed a constitutive expression, whereas *AtTPS2, AtTPS3, AtTPS4 *and *AtTPS5 *are preferentially expressed during senescence. Also, *AtTRE1 *gene showed a maximum expression during senescence. Figure 5B shows the overall expression levels of *TPS *genes and *AtTRE1 *in different *A. thaliana *organs, and led us to classify their expression in groups: 1) *AtTPS1*, *AtTPS6*, *AtTPS7*, *AtTPS8 *and *AtTPS11 *genes which are expressed upon several conditions although their highest level is in flower; 2) *AtTPS2*, *AtTPS3*, *AtTPS4 *and *AtTPS5 *genes are expressed in seeds and at very low levels in the rest of the tissues; and 3) *AtTPS9 *and *AtTPS10 *genes expressed mainly in roots. The expression of *AtTRE1 *was localized in flowers, particularly in sepals. Thus, it is likely that they have particular functions during the different plant growth stages.

### Trehalose and carbohydrate metabolism

When and how the trehalose metabolism gained extra functions is uncertain but there are several good examples of additional roles of trehalose in bacteria, yeast and plants. In *Corynebacterium glutamicum*, trehalose is a constitutive part of cell wall [62]. In *S. cerevisiae*, T6P and TPS1 have a regulatory role in carbon metabolism, and trehalose is also and storage carbohydrate [17-20]. It has been shown that trehalose in plants regulates carbon metabolism, and signals hormone and stress responses [25,58]. Nevertheless, sucrose is the most abundant free disaccharide in plant cytoplasm, and similarly to trehalose it is a non-reducing disaccharide, which in some organisms can function as an efficient osmoprotectant [63].

The synthesis of sucrose is in many aspects similar to trehalose biosynthesis. In a first step sucrose phosphate synthase (SPS) forms sucrose-6-phosphate, that is converted by sucrose phosphatase (SPP) to sucrose. These enzymes are coded by separated genes in filamentous cyanobacteria (as TPS and TPP in prokaryotes). In plants and some non-filamentous cyanobacteria, SPS has an SPP-like domain, but there is also a separate SPP protein, similar to class III TPP proteins [64,65]. In other, non-filamentous cyanobacteria there is an SPS without an SPP-like domain and a separate SPP enzyme. However, in contrast to trehalose, sucrose biosynthesis is practically limited to plants and cyanobacteria. This enormous difference in distribution between trehalose and sucrose biosynthetic genes indicates the specialisation of each of these disaccharides. It has been speculated that sucrose similarly to trehalose, was initially an osmoprotector, probably in proteobacteria, or in a common ancestor to proteobacteria and cyanobacteria. Later on, it was adopted as a metabolite for energy and carbon transport in filamentous cyanobaceria and acquired by eukaryotic cells during the endosymbiosis process from an ancestor able to synthesize sucrose [63,64]. Only plants among eukaryotes have sucrose anabolism, suggesting that the ancestral cyanobacterial endosymbiont that led to the chloroplast must have had sucrose synthesizing ability that was transferred to the host [64].

Given that the biosynthesis of trehalose is widely distributed in all domains of life, it is probably much older than sucrose biosynthesis. However, evolution adopted sucrose as the main transport and reserve carbohydrate in plants, leaving trehalose and its intermediate T6P regulatory and signalling roles. One possible explanation for the higher concentration of sucrose rather than trehalose in photosynthetic organisms is that the bond energy of sucrose is 27 kcal/mol whereas for trehalose is -1 kcal/mol, which makes the former sugar more susceptible for degradation to obtain energy [66]. Plants have a significantly higher number of enzymes involved in carbohydrate metabolism than any other organism, and it has been proposed that gene duplication during evolution of glycosyl-transferase and glycosyl-hydrolase scaffolds has allowed their vast specialization [67]. This is probably the case for *TPS *multi-gene family in plants, which one of its products, an ancient osmoprotectant, has derived in other complex functions related to growth and developmental control.

## Conclusion

We have demonstrated that trehalose biosynthetic pathways are widely distributed in nature. From the five known pathways to synthesize trehalose, the TPS/TPP route is the most conserved. Interestingly, several eubacterial species have multiple pathways, while eukaryotes have only the TPS/TPP pathway. Vertebrates have lost the capacity to synthesise trehalose but can break it down to glucose with trehalase. TPS and TPP domains have mainly evolved in parallel and it is likely that they have experienced several gene duplication and lateral gene transfer events. Some TPS and TPP sequences studied here lack functional relevant active site residues. The rate of non-synonymous to synonymous substitution of these proteins indicates that they are under selective pressure and therefore they must have a function although it is not necessarily osmoprotection. The expression pattern of the 11 *AtTPS *genes in *Arabidopsis *shows that they are expressed in a developmentally programmed and tissue-specific manner, implying a relevant function in cell metabolism.

## Methods

### Databases

The nucleotide and protein sequences of 205 completely sequenced genomes were obtained from NCBI and were explored through their online services.

### Preparation of the queries

The strategy to search for related sequences, consisted of selecting protein sequences from each one of the trehalose pathways and identify their orthologs in the completely sequenced genomes. The TPS and TPP domain sequences were identified and aligned using CLUSTAL_X [52]. The alignment was manually edited to maintain only conserved regions and deleting non-homologous sequences. The alignment edition was performed with Seaview program [53].

### Blast search

To detect homologous protein sequences from each biosynthetic pathway, we used previously reported known sequences: *E. coli *TPS [GenBank:16129848], *E. coli *TPP [GenBank:16129849], *S. avermitilis *TS [GenBank:29829345], *S. coelicolor *TreY [GenBank:21224410], *R. palustris *TreZ [GenBank:39936708], *P. furiosus *TreT [GenBank:18978114] and *P. acnes *TreP [GenBank:50842587]. The BLASTP program was used and sequences with a minimum E value of 0.0001 without filter recovered.

To identify trehalose biosynthetic genes with high accuracy in these organisms we performed BLASTP searches using the BLOSUM62 matrix, selecting only complete sequences with E^- ^values lower than 10^-9^.

### Phylogenetic tree construction

To estimate the phylogenetic relationships of the sequences we performed distant based, parsimony, and maximum likelihood analyses using the Neighbour Joining, Protpars and PHYML programs as implemented in CLUSTAL_X [52], PHYLIP [68], and PHYML [69] packages, respectively. For each method we performed bootstrapping with 1000 repetitions. The three methods gave similar clustering. Bootstrapping with 1000 resampling analyses and the corresponding consensus trees were obtained using the SEQBOOT and CONSENSE programs of the PHYLIP package [68].

### Gene neighbourhood

To determine the neighbourhood of the genes encoding TPS and TPP domains, we used the Gene Context Tool [48].

### Rate of codon substitutions

To calculate the rate of codon substitutions, the amino acid sequences of TPS and TPP alignments were reverse translated to their DNA sequence using the program JEMBOS 2.7.1 from EMBOS package, afterwards the SLAC from Datamonkey server and the PAML CODEML program [46] were used to determine the dN-dS values for each position.

### Microarray gene expression analysis

The expression patterns of *AtTPS *genes were determined using the Meta-Analizer program from Genevestigator program [61].

## Abbreviations

T6P: trehalose 6-phosphate

TPP: trehalose-phosphatase

TPS: trehalose-6-phosphate synthase

TreP: trehalose phosphorylase

TreT: trehalose glicosyltransferring synthase

TreZ: maltooligosyl trehalose synthase

TreY: maltooligosyl trehalose trehalohydrolase

TS: trehalose synthase

## Authors' contributions

NA and AMV conducted the data mining, performed the multiple alignments and tree constructions and contributed to writing the manuscript. EM supervised the study and contributed to writing the manuscript. GI conceived the study and drafted the manuscript. All authors read and approved the final manuscript.

## Supplementary Material

Additional file 1 List of the trehalose biosynthetic proteins detected in the complete sequenced organism. The taxonomic groups are shown on the left side; the accession numbers for each protein are indicated. Colours show each different protein type. Click here for file

Additional file 2 Multiple alignment of TPS domains. The alignment was performed with CLUSTAL_X [32] and edited with the Seaview program [66]. The black arrows show the residues involved in the binding to glucose-6-phosphate. The black with white arrows show the residues that bind the UDP. The box shaded indicates highly conserved regions. Click here for file

Additional file 3 Conservation of the OtsA active site along the evolution. All the proteins that conserved the complete active site of OtsA protein are labelled in blue. Click here for file

Additional file 4 Multiple alignment of TPP domains. The alignment was performed with CLUSTAL_X [32] and edited with the Seaview program [66]. The black arrows show the active site residues in HAD superfamily. The shaded box indicates highly conserved regions. Click here for file

Additional file 5 Conservation of the phosphatase consensus region along the evolution. All the proteins that conserved the complete active site are labelled in blue. Click here for file

Additional file 6 Mutational substitution rate in TPS and TPP domains. The graphics show the difference between the non-synonymous (*dN*) and the synonymous (*dS*) substitution per codon (*w *= *dN-dS*). (A) TPS. (B) TPP. Click here for file

Additional file 7 Genomic context of TPP and TPS domains. The TPS and TPP coding genes are neighbours in the genome, likely belonging to a single operon, are shown in orange. The TPS-TPP fusion proteins are labelled in black. Click here for file

Additional file 8 Phylogenetic relationship between TPS and TPP domains. The TPS domains closely related to TPP domains according the genome context are label in yellow; TPS-TPP fusion proteins are labelled in black. Click here for file
