# Supplementary material for: Insights on the evolution of trehalose biosynthesis
Source: BMC Evol Biol. 2006 Dec 19;6:109. doi: 10.1186/1471-2148-6-109 (PMC1769515; doi:10.1186/1471-2148-6-109)
Supplement: Additional file 1 — List of the trehalose biosynthetic proteins detected in the complete sequenced organism. The taxonomic groups are shown on the left side; the accession numbers for each protein are indicated. Colours show each different protein type. [file 1471-2148-6-109-S1.pdf]

	SPECIES	TPS	TPP	TS	TREY	TREZ	TRET	TREP
Archaea	*M.thermautotrophicus*	GI:15679745	GI:15679748					
	P.aerophilum	GI:18312516					GI:18312520	
	P.furiosus						GI:18978114	
	P.horikoshii						GI:14590874	GI:14590619
	S.solfataricus				GI:15898879	GI:15898877	GI:15899335	
	S.tokodaii				GI:15921163		GI:15920609	
	T.acidophilum	GI:16082218	GI:16082598					
	T.volcanium	GI:13542085	GI:13542086					
Bacteria	*A.tumefaciens*					GI:16119513		
	B.licheniformis							GI:52079099
	B.subtilis							GI:16080510
	B.fragilis							GI:53714596
	B.bronchiseptica			GI:33601839	GI:33601844	GI:33601842		
	B.pertussis			GI:33592443	GI:33592439	GI:33592440		
	B.japonicum	GI:27375433	GI:27375434	GI:27381878	GI:27381882	GI:27381881		
	C.tepidum			GI:21674898				GI:21673667
	C.diphtheriae	GI:38234530	GI:38234532					
	C.efficiens	GI:25029064	GI:25029066	GI:25028761	GI:25028574	GI:25028581		
	C.glutamicum	GI:19553823	GI:19553825	GI:23308924	GI:19553316	GI:23308896		
	D.radiodurans			GI:15807030	GI:15805490	GI:15805491		
	E.faecalis							GI:29375540
	E.coli	GI:16129848	GI:16129849					GI:16129277
	G.sulfurreducens	GI:39997434	GI:39997433	GI:39997457	GI:39997456	GI:39997454		
	L.johnsonii							GI:42518299
	L.plantarum							GI:28379843
	L.lactis							GI:15673659
	M.loti _1	GI:13470877	GI:13470876					
	M.loti _2	GI:13488444						
	M. avium	GI:41406671	GI:41409576	GI:41409626	GI:41407367	GI:41407366		GI:41409590
	M.bovis	GI:31794666	GI:31793186			GI:31792747		GI:31794582
	M.leprae	GI:15828204	GI:15827129					GI:15827117
	M.tuberculosis _1	GI:15610626	GI:15842968	GI:15607268	GI:15608701	GI:15841029		GI:15842995
	M.tuberculosis _2	GI:15843102						
	N. meningitidis							GI:15676304
	Nostoc sp.				GI:17227663	GI:17227664		GI:17228553
	Parachlamydia sp.				GI:46447190			
	Pirellula sp.					GI:32473570		
	P.acnes	GI: 50842445		GI:50842592				GI:50842587
	P.aeruginosa			GI:15597348		GI:15597360		
	P.putida			GI:26990760	GI:26990754	GI:26990752		
	P.syringae			GI:28869947	GI:28870301	GI:28870299		
	R.solanacearum _1	GI:17549326	GI:17549325	GI:17548461	GI:17548456	GI:17548458		
	R.solanacearum _2	GI:17548952						
	R.palustris	GI:39937718	GI:39937719	GI:39936705	GI:39936709	GI:39936708		
	S.enterica	GI:16760877	GI:16760878		GI:16760316	GI:16760315		
	S.typhimurium	GI:16765270	GI:16765271		GI:16764903	GI:16764904		
	S.flexneri		GI:30063346					GI:30062835
	S.meliloti	GI:16262578			GI:16265234	GI:16265024		
	S.avermitilis	GI:29830479	GI:29830480	GI:29829345	GI:29828694	GI:29828701		

	SPECIES	TPS	TPP	TS	TREY	TREZ	TRET	TREP
	S.coelicolor	GI:21222683	GI:21222681	GI:21223801	GI:21224410	GI:21224407		GI:21219247
	Synechococcus sp.	GI:3152388						
	Synechocystis sp.	GI:16330944						
	T.maritima						GI:15643158	
	T.tengcongensis _1							GI:20807289
	T.tengcongensis _2							GI:20807280
	X.axonopodis	GI:21243937	GI:21243935	GI:21240929	GI:21241202	GI:21241200		
	X.campestris _1	GI:21232512	GI:21232510	GI:21229612	GI:21229889	GI:21229887		
	X.campestris _2	GI:21232465						
Fungi	*E.cuniculi*	GI:19075090	GI:19075097					
	E.gossypii _1	GI:45187495						
	E.gossypii _2	GI:45201228						
	E. gossypii _3	GI:45190879						
	S.cerevisiae ScTPS2	GI:6320279						
	S.cerevisiae ScTPS3	GI:6323917						
	S.cerevisiae ScTSL1	GI:6323537						
	S.cerevisiae _1	GI:6319602						
	S.pombe _1 SpTPS1	GI:19115117						
	S.pombe _2 SpTPS2	GI:19115887						
	S.pombe _3 SpTPS3	GI:19115342						
	S. pombe _4 SpTPS4	GI:19114874						
	S. pombe _5 SpTPS5	GI:19115640						
Metazoa	*A.gambiae*	GI:31222942						
	C.elegans (1)	GI:32563851						
	C.elegans (2)	GI:25147603						
	H.sapiens							
	M.musculus							
	R.norvegicus							
	D.melanogaster	GI:19920676						
Plantae	*A.thaliana* _1 AtTPS1	GI:15218422						
	A.thaliana _2 AtTPS2	GI:15219969						
	A.thaliana _3 AtTPS3	GI:15219985						
	A.thaliana _4 AtTPS4	GI:15234194						
	A.thaliana _5 AtTPS5	GI:18414960						
	A.thaliana _6 AtTPS6	GI:12324075						
	A.thaliana _7 AtTPS7	GI:15221478						
	A.thaliana _8 AtTPS8	GI:30698024						
	A.thaliana _9 AtTPS9	GI:15220891						
	A.thaliana _10 AtTPS10	GI:15219002						
	A.thaliana _11 AtTPS11	GI:15224213						
	A.thaliana _21 AtTPPA		GI:42573652					
	A.thaliana _22 AtTPPB		GI:15218205					
	A.thaliana _20 AtTPPC		GI:15219843					
	A.thaliana _19 AtTPPD		GI:18399940					
	A.thaliana _18 AtTPPE		GI:15227161					
	A.thaliana _17 AtTPPF		GI:15234552					
	A.thaliana _16 AtTPPG		GI:15235713					
	A.thaliana _14 AtTPPH		GI:30692524					
	A.thaliana _13 AtTPPI		GI:30683008					
	A.thaliana _12 AtTPPJ		GI:42568786					
	O.sativa _1 OsTPS1	GI:34910846						
	O.sativa _2 OsTPS2	GI:34902280						
	O.sativa _3 OsTPS3	GI:34909526						
	O.sativa _4 OsTPS4	GI:37806209						
	O.sativa _5 OsTPS5	GI:42408334						
	O.sativa _6 OsTPPA		GI:37536726					
	O.sativa _7 OsTPPB		GI:33146623					
	O.sativa _8 OsTPPC		GI:37806433					
	O.sativa _9 OsTPPD		GI:38345480					
	O.sativa _10 OsTPPE		GI:45544517					
	O.sativa _11 OsTPPF		GI:46390128					
