# Supplementary material for: Insights on the evolution of trehalose biosynthesis
Source: BMC Evol Biol. 2006 Dec 19;6:109. doi: 10.1186/1471-2148-6-109 (PMC1769515; doi:10.1186/1471-2148-6-109)
Supplement: Additional file 2 — Multiple alignment of TPS domains. The alignment was performed with CLUSTAL_X [32] and edited with the Seaview program [66]. The black arrows show the residues involved in the binding to glucose-6-phosphate. The black with white arrows show the residues that bind the UDP. The box shaded indicates highly conserved regions. [file 1471-2148-6-109-S2.pdf]

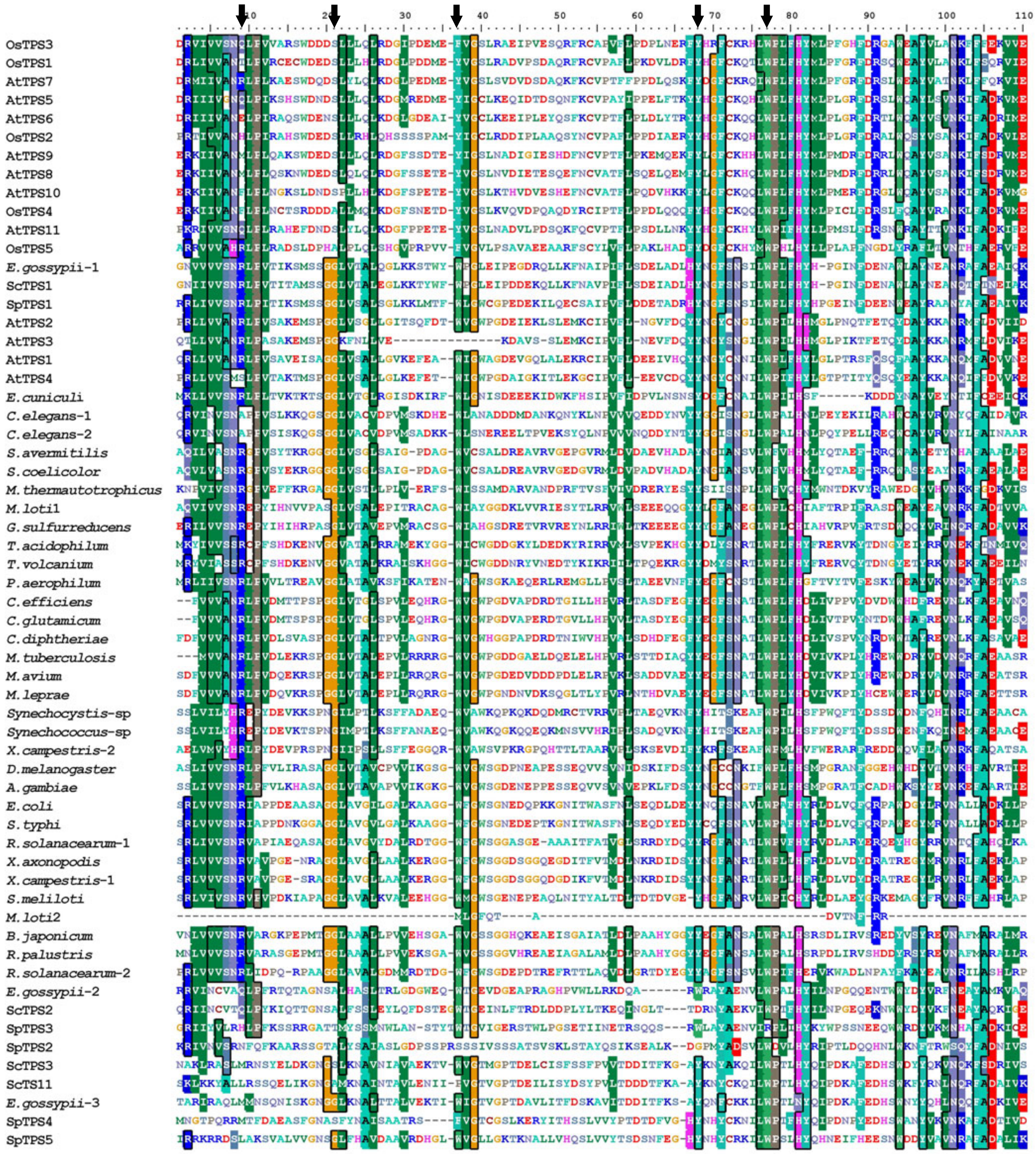
10
20
30
40
50
60
70
80
90
100
110
OsTPS3
OsTPS1
AtTPS7
AtTPS5
AtTPS6
OsTPS2
AtTPS9
AtTPS8
AtTPS10
OsTPS4
AtTPS11
OsTPS5
E.gossypii-1
ScTPS1
SpTPS1
AtTPS2
AtTPS3
AtTPS1
AtTPS4
E.cuniculi
C.elegans-1
C.elegans-2
S.avermitilis
S.coelicolor
M.thermautotrophicus
M.loti1
G.sulfurreducens
T.acidophilum
T.volcanium
P.aerophilum
C.efficiens
C.glutamicum
C.diphtheriae
M.tuberculosis
M.avium
M.leprae
Synechocystis-sp
Synechococcus-sp
X.campestris-2
D.melanogaster
A.gambiae
E.coli
S.typhi
R.solanacearum-1
X.axonopodis
X.campestris-1
S.meliloti
M.loti2
B.japonicum
R.palustris
R.solanacearum-2
E.gossypii-2
ScTPS2
SpTPS3
SpTPS2
ScTPS3
ScTS11
E.gossypii-3
SpTPS4
SpTPS5

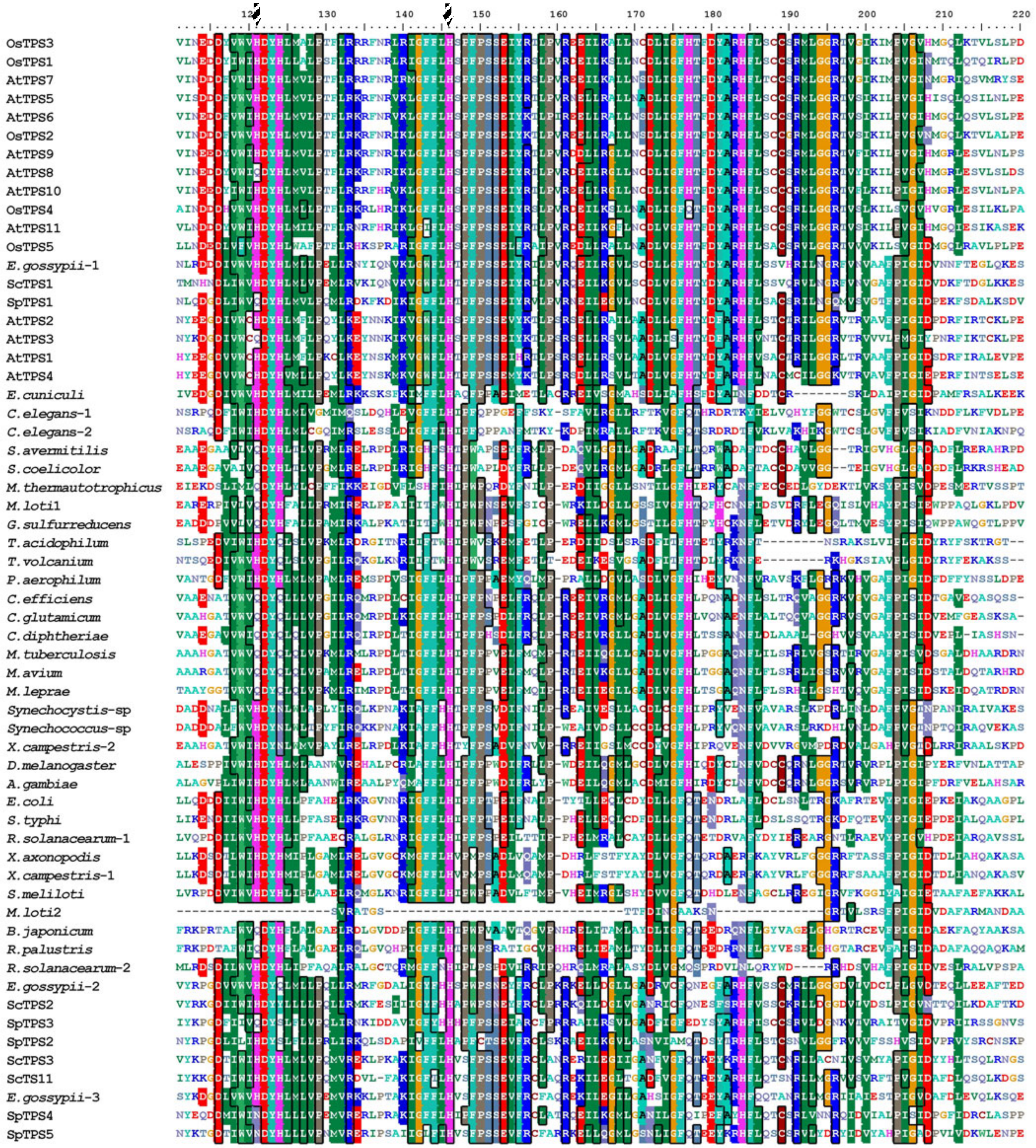
120
130
140
150
160
170
180
190
200
210
220
OsTPS3
OsTPS1
AtTPS7
AtTPS5
AtTPS6
OsTPS2
AtTPS9
AtTPS8
AtTPS10
OsTPS4
AtTPS11
OsTPS5
E.gossypii-1
ScTPS1
SpTPS1
AtTPS2
AtTPS3
AtTPS1
AtTPS4
E.cuniculi
C.elegans-1
C.elegans-2
S.avermitilis
S.coelicolor
M.thermautotrophicus
M.loti1
G.sulfurreducens
T.acidophilum
T.volcanium
P.aerophilum
C.efficiens
C.glutamicum
C.diphtheriae
M.tuberculosis
M.avium
M.leprae
Synechocystis-sp
Synechococcus-sp
X.campestris-2
D.melanogaster
A.gambiae
E.coli
S.typhi
R.solanacearum-1
X.axonopodis
X.campestris-1
S.meliloti
M.loti2
B.japonicum
R.palustris
R.solanacearum-2
E.gossypii-2
ScTPS2
SpTPS3
SpTPS2
ScTPS3
ScTS11
E.gossypii-3
SpTPS4
SpTPS5

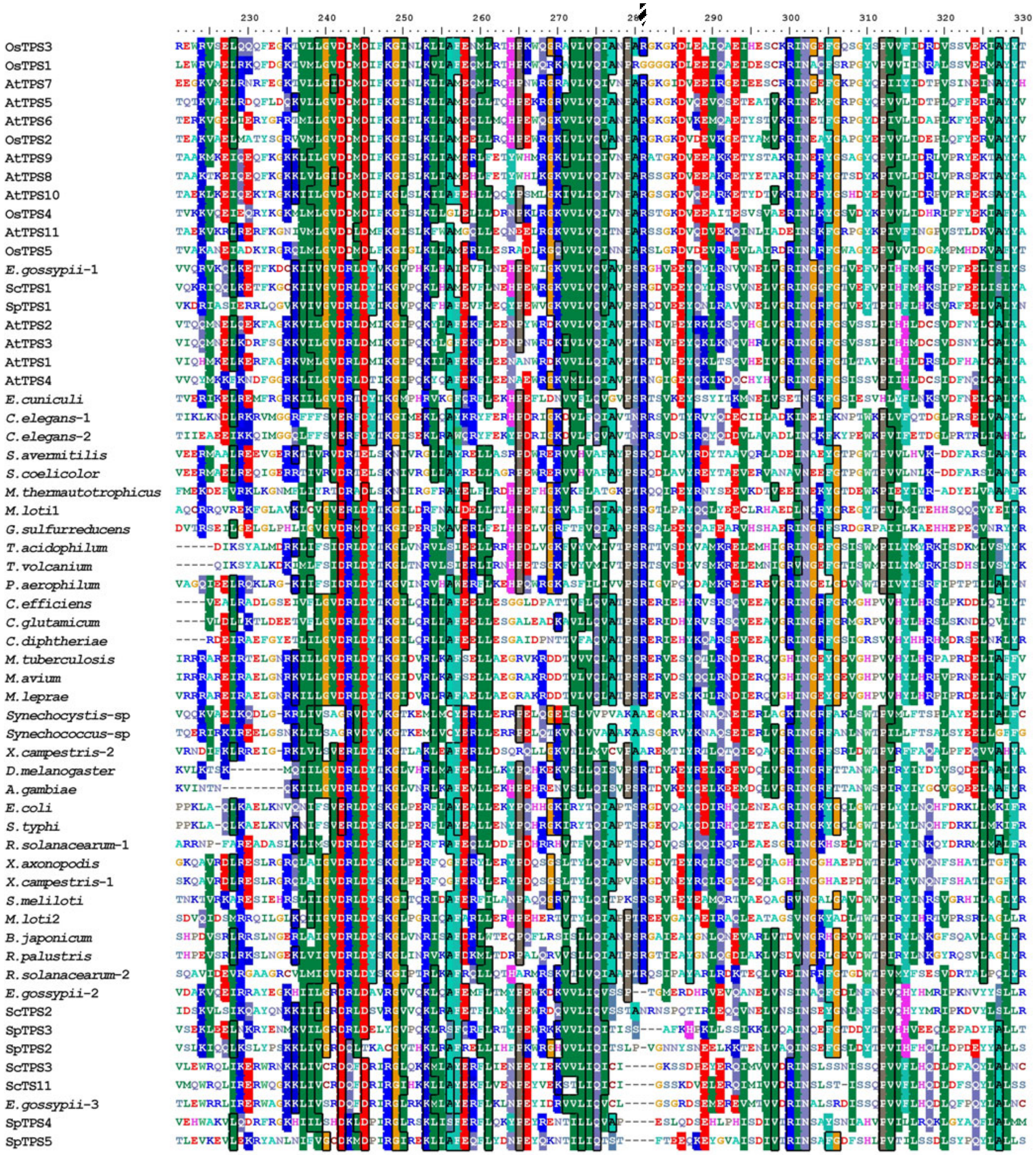
230
240
250
260
270
280
290
300
310
320
330
OsTPS3
OsTPS1
AtTPS7
AtTPS5
AtTPS6
OsTPS2
AtTPS9
AtTPS8
AtTPS10
OsTPS4
AtTPS11
OsTPS5
E.gossypii-1
ScTPS1
SpTPS1
AtTPS2
AtTPS3
AtTPS1
AtTPS4
E.cuniculi
C.elegans-1
C.elegans-2
S.avermitilis
S.coelicolor
M.thermautotrophicus
M.loti1
G.sulfurreducens
T.acidophilum
T.volcanium
P.aerophilum
C.efficiens
C.glutamicum
C.diphtheriae
M.tuberculosis
M.avium
M.leprae
Synechocystis-sp
Synechococcus-sp
X.campestris-2
D.melanogaster
A.gambiae
E.coli
S.typhi
R.solanacearum-1
X.axonopodis
X.campestris-1
S.meliloti
M.loti2
B.japonicum
R.palustris
R.solanacearum-2
E.gossypii-2
ScTPS2
SpTPS3
SpTPS2
ScTPS3
ScTS11
E.gossypii-3
SpTPS4
SpTPS5

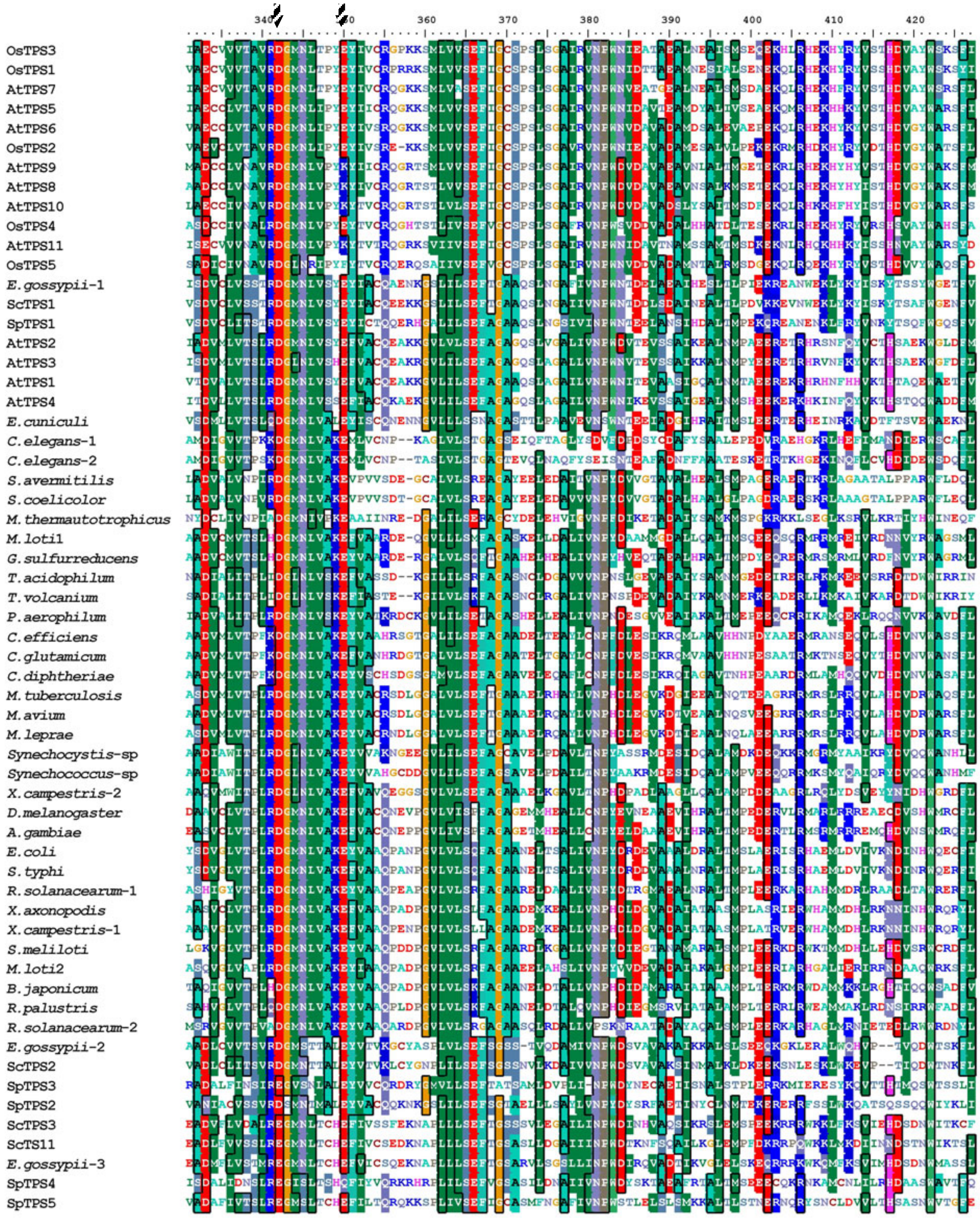
340
360
370
380
390
400
410
420
OsTPS3
OsTPS1
AtTPS7
AtTPS5
AtTPS6
OsTPS2
AtTPS9
AtTPS8
AtTPS10
OsTPS4
AtTPS11
OsTPS5
E.gossypii-1
ScTPS1
SpTPS1
AtTPS2
AtTPS3
AtTPS1
AtTPS4
E.cuniculi
C.elegans-1
C.elegans-2
S.avermitilis
S.coelicolor
M.thermautotrophicus
M.loti1
G.sulfurreducens
T.acidophilum
T.volcanium
P.aerophilum
C.efficiens
C.glutamicum
C.diphtheriae
M.tuberculosis
M.avium
M.leprae
Synechocystis-sp
Synechococcus-sp
X.campestris-2
D.melanogaster
A.gambiae
E.coli
S.typhi
R.solanacearum-1
X.axonopodis
X.campestris-1
S.meliloti
M.loti2
B.japonicum
R.palustris
R.solanacearum-2
E.gossypii-2
ScTPS2
SpTPS3
SpTPS2
ScTPS3
ScTS11
E.gossypii-3
SpTPS4
SpTPS5
