# Supplementary figures and images for: Insights on the evolution of trehalose biosynthesis
Source: BMC Evol Biol. 2006 Dec 19;6:109. doi: 10.1186/1471-2148-6-109 (PMC1769515; doi:10.1186/1471-2148-6-109)

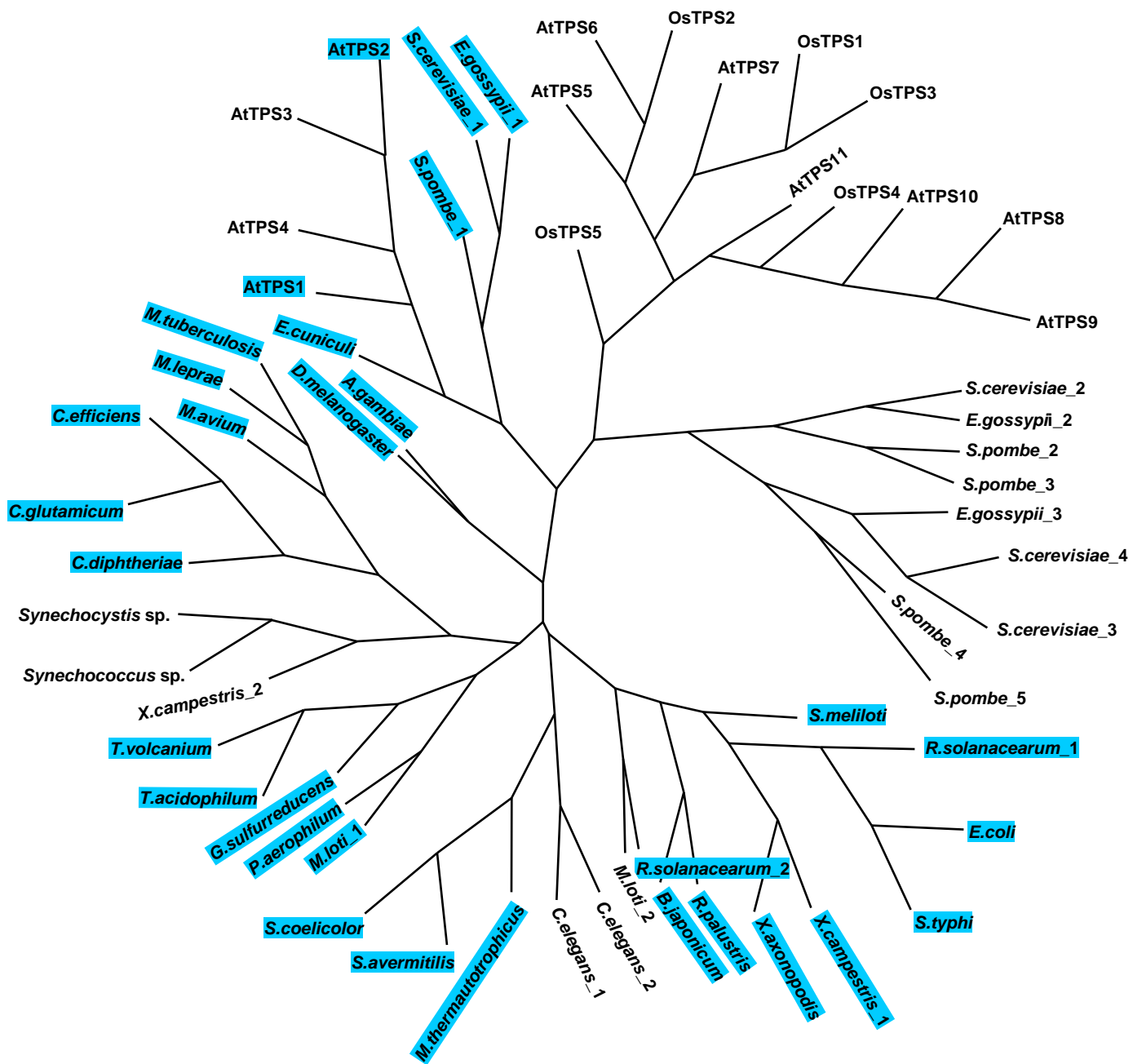

Supplement: Additional file 3 — Conservation of the OtsA active site along the evolution. All the proteins that conserved the complete active site of OtsA protein are labelled in blue. [file 1471-2148-6-109-S3.pdf]

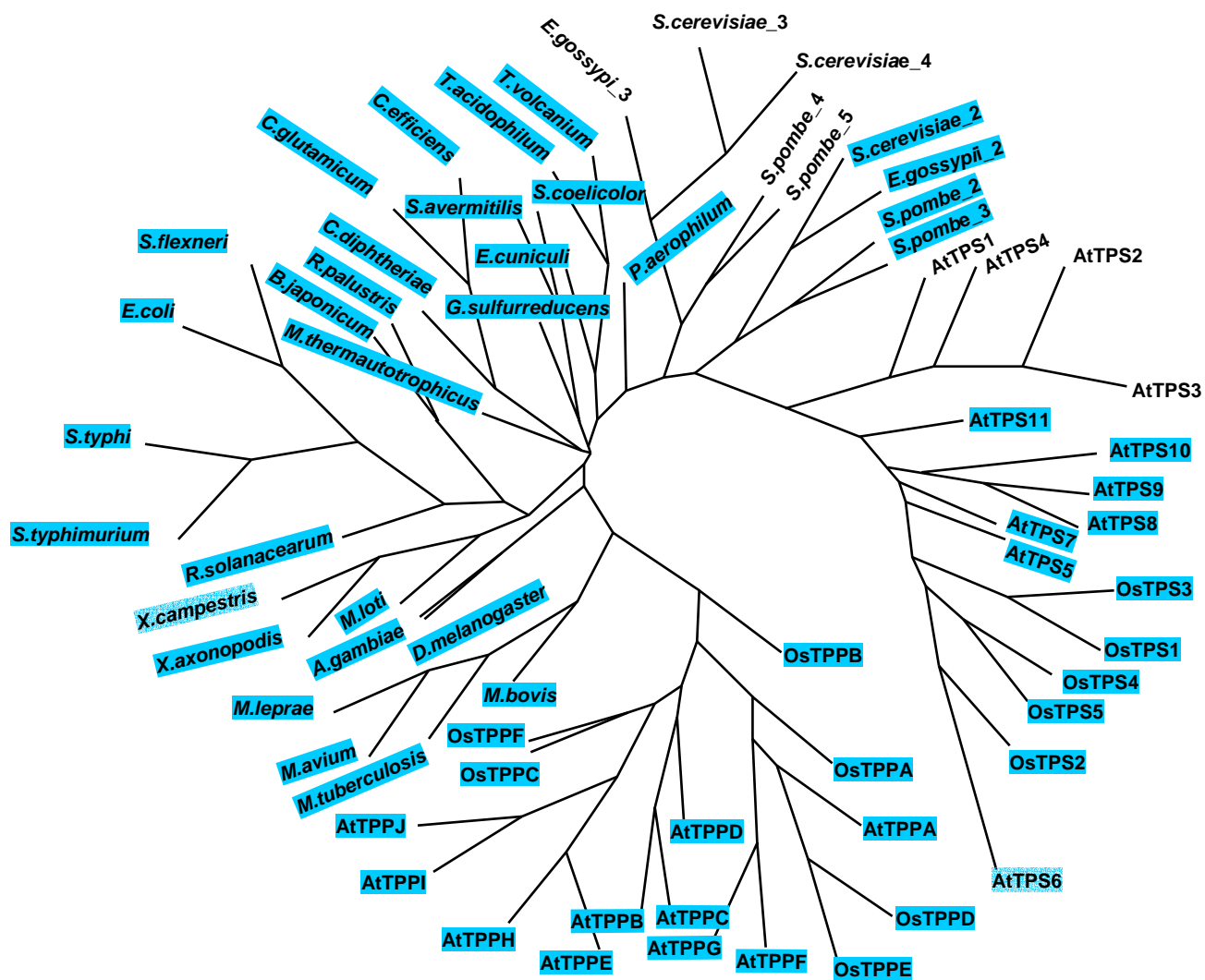

Supplement: Additional file 5 — Conservation of the phosphatase consensus region along the evolution. All the proteins that conserved the complete active site are labelled in blue. [file 1471-2148-6-109-S5.pdf]

**A)**

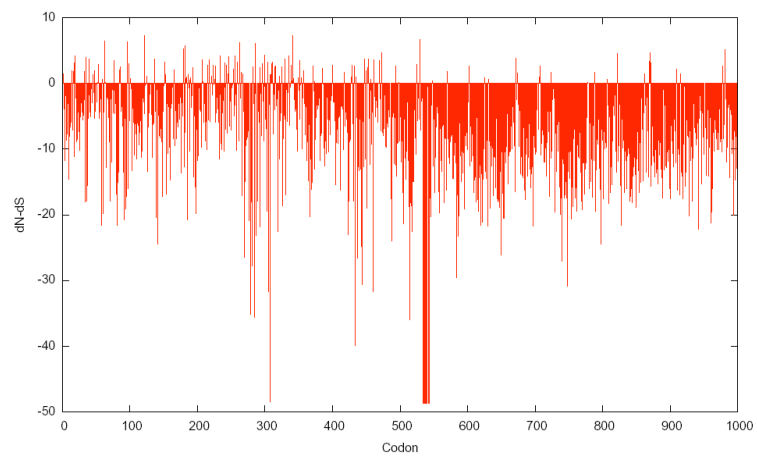

**B)**

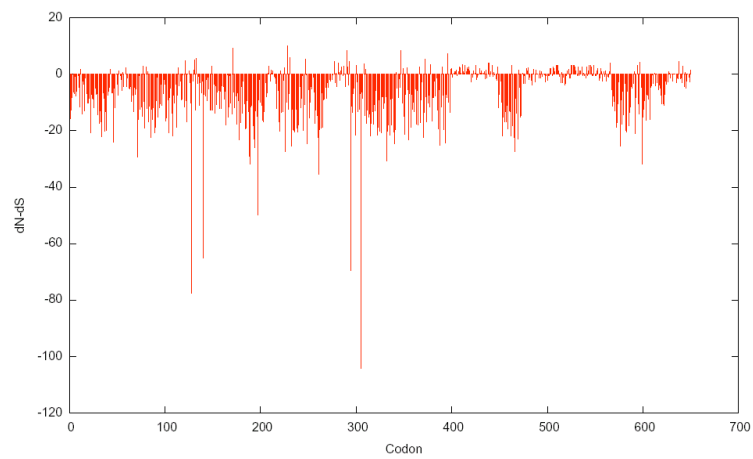

Supplement: Additional file 6 — Mutational substitution rate in TPS and TPP domains. The graphics show the difference between the non-synonymous (dN) and the synonymous (dS) substitution per codon (w = dN-dS). (A) TPS. (B) TPP. [file 1471-2148-6-109-S6.pdf]

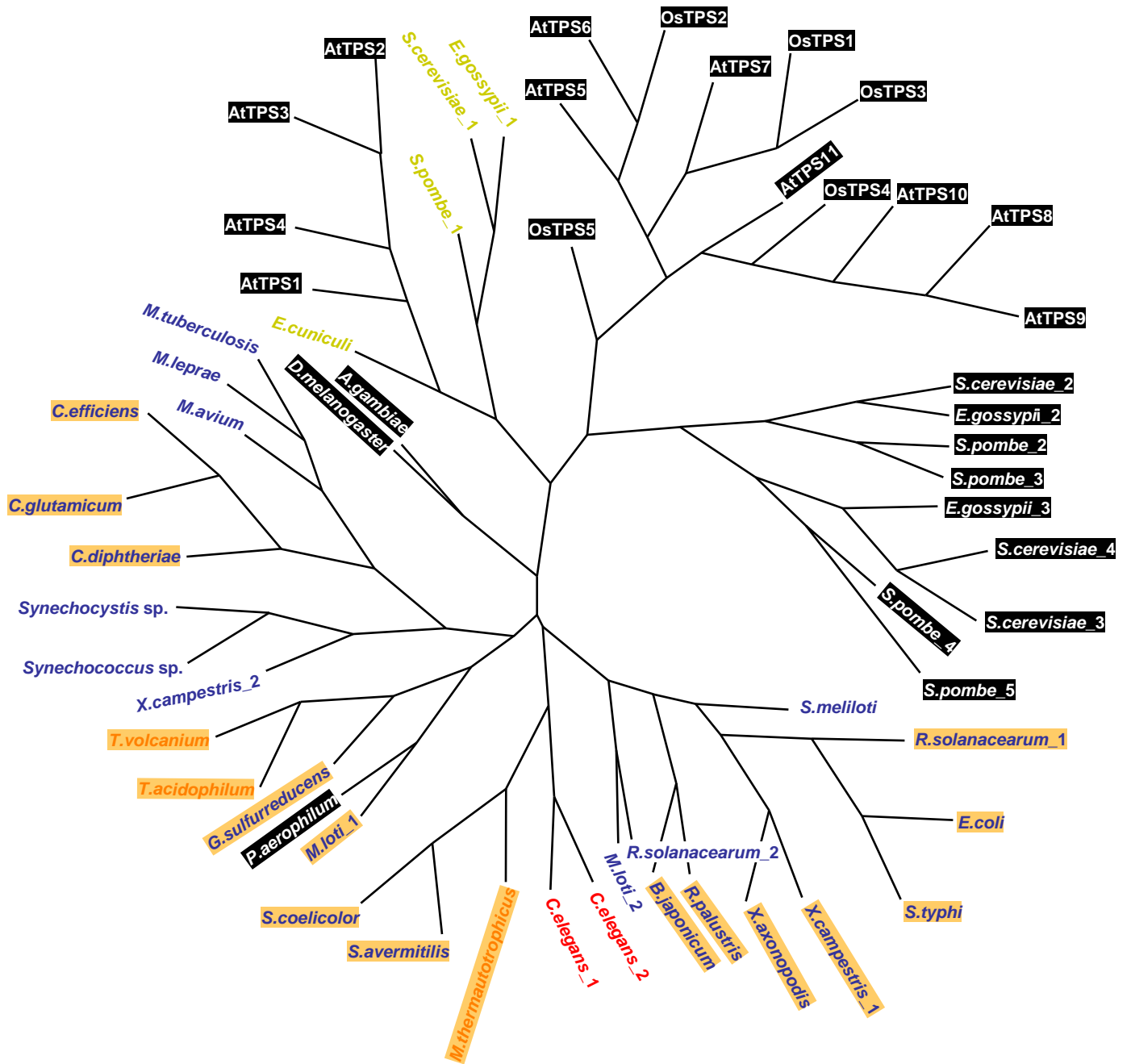

Supplement: Additional file 7 — Genomic context of TPP and TPS domains. The TPS and TPP coding genes are neighbours in the genome, likely belonging to a single operon, are shown in orange. The TPS-TPP fusion proteins are labelled in black. [file 1471-2148-6-109-S7.pdf]

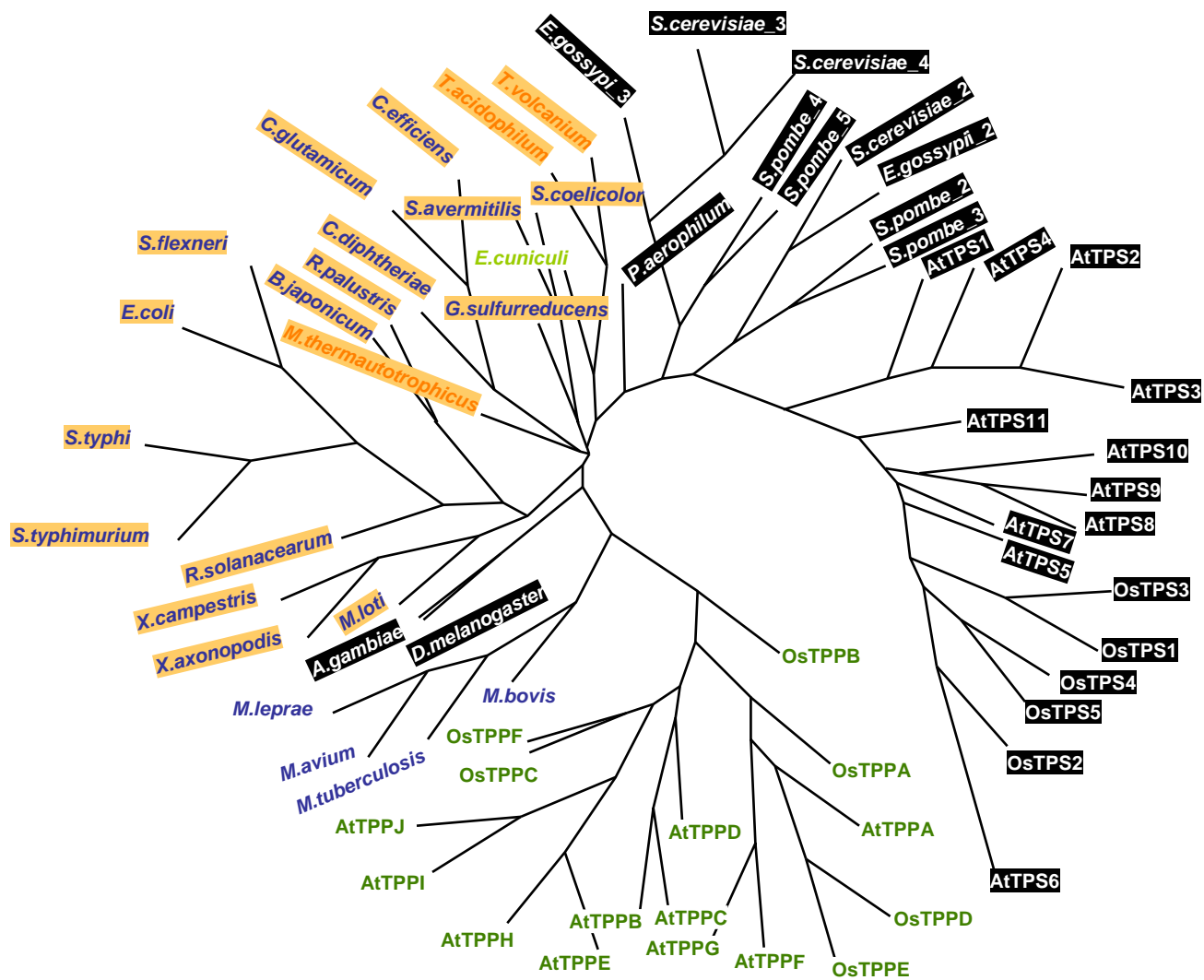

Supplement: Additional file 8 — Phylogenetic relationship between TPS and TPP domains. The TPS domains closely related to TPP domains according the genome context are label in yellow; TPS-TPP fusion proteins are labelled in black. [file 1471-2148-6-109-S8.pdf]
