# Supplementary material for: Insights on the evolution of trehalose biosynthesis
Source: BMC Evol Biol. 2006 Dec 19;6:109. doi: 10.1186/1471-2148-6-109 (PMC1769515; doi:10.1186/1471-2148-6-109)
Supplement: Additional file 4 — Multiple alignment of TPP domains. The alignment was performed with CLUSTAL_X [32] and edited with the Seaview program [66]. The black arrows show the active site residues in HAD superfamily. The shaded box indicates highly conserved regions. [file 1471-2148-6-109-S4.pdf]

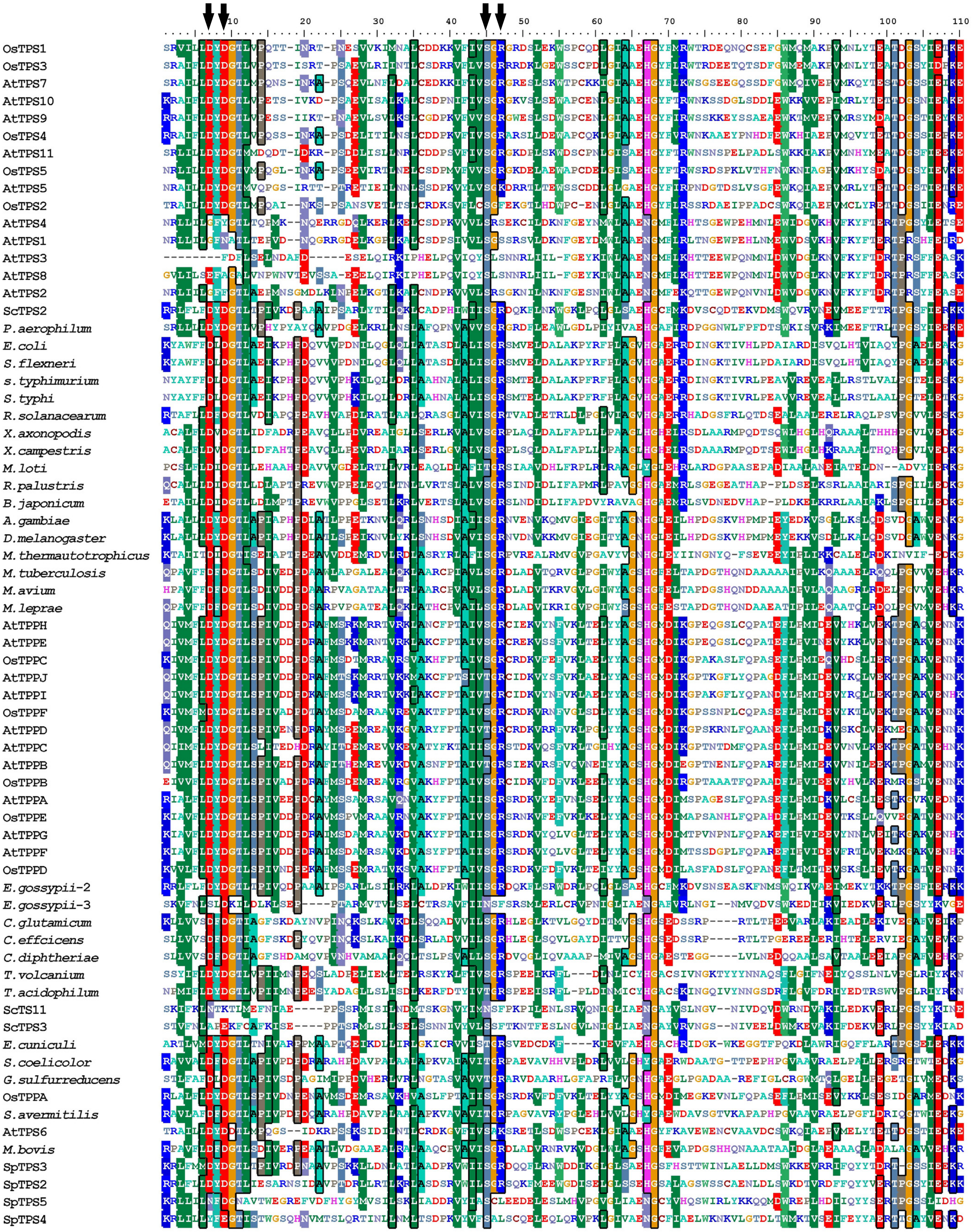

10
20
30
40
50
60
70
80
90
100
110
OsTPS1
OsTPS3
AtTPS7
AtTPS10
AtTPS9
OsTPS4
AtTPS11
OsTPS5
AtTPS5
OsTPS2
AtTPS4
AtTPS1
AtTPS3
AtTPS8
AtTPS2
ScTPS2
P.aerophilum
E.coli
S.flexneri
s.typhimurium
S.typhi
R.solanacearum
X.axonopodis
X.campestris
M.loti
R.palustris
B.japonicum
A.gambiae
D.melanogaster
M.thermautotrophicus
M.tuberculosis
M.avium
M.leprae
AtTPPH
AtTPPE
OsTPPC
AtTPPJ
AtTPPI
OsTPPF
AtTPPD
AtTPPC
AtTPPB
OsTPPB
AtTPPA
OsTPPE
AtTPPG
AtTPPF
OsTPPD
E.gossypii-2
E.gossypii-3
C.glutamicum
C.effcicens
C.diphtheriae
T.volcanium
T.acidophilum
ScTS11
ScTPS3
E.cuniculi
S.coelicolor
G.sulfurreducens
OsTPPA
S.avermitilis
AtTPS6
M.bovis
SpTPS3
SpTPS2
SpTPS5
SpTPS4

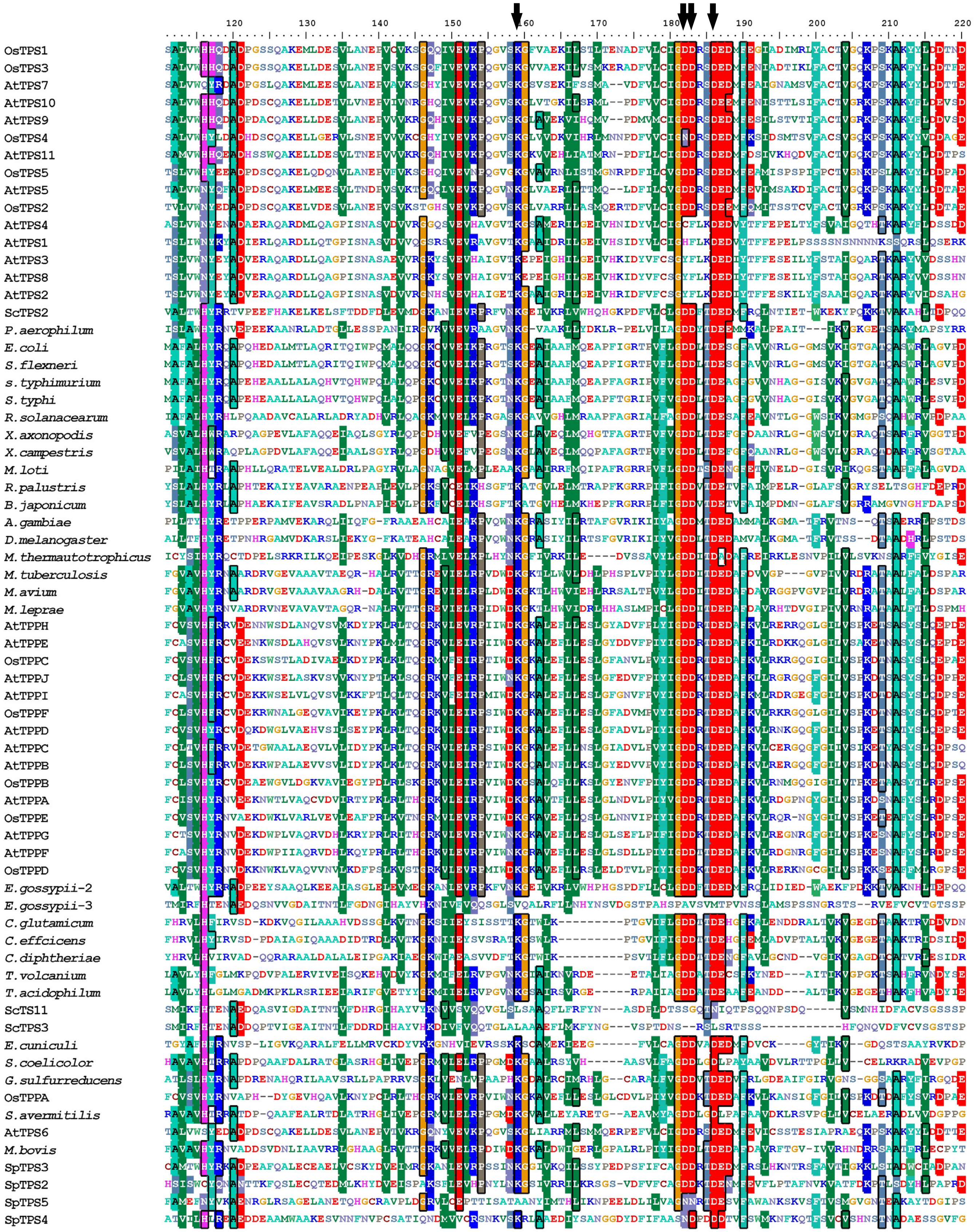
120 130 140 150 160 170 180 190 200 210 220
OsTPS1
OsTPS3
AtTPS7
AtTPS10
AtTPS9
OsTPS4
AtTPS11
OsTPS5
AtTPS5
OsTPS2
AtTPS4
AtTPS1
AtTPS3
AtTPS8
AtTPS2
ScTPS2
P.aerophilum
E.coli
S.flexneri
s.typhimurium
S.typhi
R.solanacearum
X.axonopodis
X.campestris
M.loti
R.palustris
B.japonicum
A.gambiae
D.melanogaster
M.thermautotrophicus
M.tuberculosis
M.avium
M.leprae
AtTPPH
AtTPPE
OsTPPC
AtTPPJ
AtTPPI
OsTPPF
AtTPPD
AtTPPC
AtTPPB
OsTPPB
AtTPPA
OsTPPE
AtTPPG
AtTPPF
OsTPPD
E.gossypii-2
E.gossypii-3
C.glutamicum
C.effcicens
C.diphtheriae
T.volcanium
T.acidophilum
ScTS11
ScTPS3
E.cuniculi
S.coelicolor
G.sulfurreducens
OsTPPA
S.avermitilis
AtTPS6
M.bovis
SpTPS3
SpTPS2
SpTPS5
SpTPS4

```
                          230
                     ....|....|..
OsTPS1               VLNMLEALADA-
OsTPS3               VVTMLSALADA-
AtTPS7               VT----------
AtTPS10              VV----------
AtTPS9               VL----------
OsTPS4               VVRLLKNVAGI-
AtTPS11              VI----------
OsTPS5               VVKMLQGLTDS-
AtTPS5               II----------
OsTPS2               VVRLMQGLASV-
AtTPS4               VV----------
AtTPS1               S-----------
AtTPS3               VV----------
AtTPS8               VV----------
AtTPS2               VV----------
ScTPS2               VLETLGLLVGD-
P.aerophilum         IRELLQALLTA-
E.coli               VWSWLEMITTA-
S.flexneri           VWSWLEMITAA-
s.typhimurium        VWRWLEQINYP-
S.typhi              VWRWLEQINYP-
R.solanacearum       LRDWLAALARR-
X.axonopodis         VHAWLQRNARS-
X.campestris         VHAWLQRSAHT-
M.loti               ALAWLDEMSRS-
R.palustris          VRNWLARLLEP-
B.japonicum          VRAFLARLLDP-
A.gambiae            VLTMLKWVERH-
D.melanogaster       VYTLLKWVER--
M.thermautotrophicus VLRFFRWLLK--
M.tuberculosis       VAEFTDRLARQ-
M.avium              AAEFTERLADQ-
M.leprae             VSEFTERLARQ-
AtTPPH               VMEFLQRLVEW-
AtTPPE               VMVFLERLVEW-
OsTPPC               VMEFLLRLVEW-
AtTPPJ               VMNFLGRLVEW-
AtTPPI               VMDFLRRLVEW-
OsTPPF               VMEFLLRLVEW-
AtTPPD               VGEFLQRLVEW-
AtTPPC               VKEFLERLVKW-
AtTPPB               VNKFLERLVEW-
OsTPPB               VKEFLRKLVKI-
AtTPPA               VMEFLKSLVTW-
OsTPPE               VMEFLNFLVRW-
AtTPPG               VKKFLKTLVKW-
AtTPPF               VKKFLKTLVKW-
OsTPPD               VVILPVMLNFF-
E.gossypii-2         VLDTLGLLVGD-
E.gossypii-3         VLEP--------
C.glutamicum         VGIFLEKLAYH-
C.effcicens          VGIMLEELAYE-
C.diphtheriae        VGQFLSSLADA-
T.volcanium          MREVLKLLLNN-
T.acidophilum        MRKILKFIEML-
ScTS11               VLEPLFKLVND-
ScTPS3               IIEPLFKLVKQ-
E.cuniculi           --ENFRMLLGR-
S.coelicolor         AGVVALLAALA-
G.sulfurreducens     MAPLLDEILAV-
OsTPPA               VMEFLKKLASW-
S.avermitilis        VVSLLYALAAR-
AtTPS6               IVRLMHGLASV-
M.bovis              VCQFLSQLACD-
SpTPS3               VIDILADLANF-
SpTPS2               LGELLHNL----
SpTPS5               FFNVLNSLCA--
SpTPS4               FLQALEKVYSA-
```
